# An experimental study of the dual modulation of the colchicine-induced rat model of Alzheimer’s disease by superparamagnetic iron oxide nanoparticles (SPIONs) and the soluble product of *Dipylidium caninum* adult worm

**DOI:** 10.1371/journal.pone.0324191

**Published:** 2025-06-03

**Authors:** Amal M. Elsharkawy, Faika Hassanein, Samia S. Abouelkheir, Inas M. Masoud, Wael Felefel, Inas E. Darwish

**Affiliations:** 1 Faculty of Medicine, Department of Clinical Pharmacology, Alexandria University, Alexandria, Egypt; 2 Faculty of Dentistry, Department of Microbiology and Immunology, Pharos University in Alexandria, Alexandria, Egypt; 3 National Institute of Oceanography and Fisheries (NIOF), Cairo, Egypt; 4 Faculty of Pharmacy, Department of Pharmacology and Therapeutics, Pharos University in Alexandria, Alexandria, Egypt; 5 Faculty of Veterinary Medicine, Department of Parasitology, Matrouh University, Matrouh, Egypt; Benha University Faculty of Veterinary Medicine, EGYPT

## Abstract

Alzheimer’s disease, affecting 7.24 million globally, requires combination therapies, including cholinesterase inhibitors and immunotherapy, for optimal management, emphasizing the benefits of these treatments. The current study investigated the potential synergy between superparamagnetic iron oxide nanoparticles (SPIONs) and a soluble product of *Dipylidium caninum* adult worms to enhance biochemical and cognitive changes in a colchicine-induced rat model of Alzheimer’s disease. The study involved 50 male albino rats randomly assigned into five groups: group I, the negative control; group II, the positive control; group III, the soluble product of *D. caninum* excretory-secretory product (ESP)-intervened group; group IV, the SPION-intervened group; and group V, the combination of SPIONs and the soluble product of *D. caninum* adult worm ESP-intervened group. Each group consisted of 10 rats. The study compared the biochemical and cognitive abilities of the three intervention groups to the negative control using the MANOVA test, revealing a significant fit model (p = 0.000) and a large effect size (partial eta squared = 0.750) for the biochemical improvement. The same results were found for all biochemical tests, including amyloid beta (AB1–42), nuclear factor-kappa B (NF-kB), malondialdehyde (MDA), and superoxide dismutase (SOD). The effect sizes were large (0.551, 0.729, 0.674, and 0.445, respectively), and the fit models were significant (P = 0.000). When comparing the experimental groups pairwise, it was clear that group V was the most effective therapy, as it had the smallest mean difference across all biochemical markers when compared to the negative control group. Regarding cognitive changes, both the multivariate test and tests of between-subject effects of all seven cognitive parameters were significant, with P values ≤ 0.05 and a large effect size due to the partial eta squared values above 0.1. However, for the passive avoidance (PA) effect, initial latency and locomotor activity have medium effect sizes with values between 0.01 and 0.1 (0.061 and 0.018, respectively). Pairwise comparisons between intervention and negative control groups revealed that group IV (SPION-intervened) had the smallest values of cognitive parameters, making it the best intervention therapy for cognitive changes. The Log Rank (Mantel-Cox) Kaplan-Meier curve indicated that the least mean timing needed for cognitive changes to normalize was 20 days at a median point of 0.5, with group III (*D. caninum* ESP-intervened group) having a significantly different Log Rank (Chi-Square = 85.490, P = 0.000).

## Introduction

Researchers have found that Alzheimer’s disease (AD) contributes to at least two-thirds of cognitive disorders in personal belongings among older adults. This is the most popular kind of memory loss. In the United States, it ranks as the sixth most frequent reason for death [1]. These neuropathological abnormalities incorporate extracellular amyloid plaques generated by Aβ peptide accumulation from the amyloid precursor protein cleavage, in addition to intracellular tau protein-based neurofibrillary tangles [2]. Globally, there were 7.24 million cases of AD in 2019 [3], suggesting an immense rise in the prevalence of AD and other dementias over time. One percent of older adults in Upper Egypt had AD [4]. A mortality frequency of 37 deaths per 100,000 people is associated with AD [5,6]. Most people accept the amyloid cascade theory as the most plausible explanation for AD [7]. However, the current failure of anti-amyloid remedies has raised questions about the sequential order of pathogenic processes in AD [8]. It is difficult to attract volunteers for therapy research since the evidence regarding the neuropathological variations in AD is not always consistent. This discrepancy may lead to intervention disappointments [9].

The rate of medication failure for AD is high [10], and despite the study of numerous compounds in phase III trials [11], no new disease-modifying treatments for AD became available in 2019. However, in terms of drug-target engagement and brain amyloid-β clearance, these trials did yield positive results [10]. The “Hygiene Hypothesis” asserts that infancy contact with infectious agents is vital for immune system development [12]. Intestinal helminths, rare in their ability to activate immunoregulatory pathways, could potentially treat chronic inflammatory disorders [13,14]. Given the failure of Aβ-targeting treatment trials, it is imperative that new theories are explored and new intervention goals are developed for AD [15]. Regulatory T cells (Tregs) are primarily responsible for suppressing inflammatory and autoimmune responses [16,17].

Research has demonstrated that helminthic treatment can significantly reduce autoimmune responses by reducing Th1 and Th17 cells and increasing Th2 cells, Tregs, and regulatory macrophages [18]. *Dipylidium caninum* is a cestode that belongs to the order Cyclophyllidea and family Dipylidiidae [19]. Furthermore, it’s feasible that a person’s illness is self-limiting and has an innate healing mechanism [20]. Adult *D. caninum* worms consume food in the small intestine and aggressively release excretory-secretory products (ESP) into the mucosa to enhance their living environment and prevent allergic reactions from the host [21]. Researchers have found that the *D. caninum* ESP reduces inflammation by activating regulatory B cells [22], tolerogenic dendritic cells [23], and regulatory T cells [24]. These cells create suppressor cytokines that inhibit the inflammatory T cells and their mediator proteins. A higher molecular mass preparation of the rat tapeworm *Hymenolepis diminuta* can protect rat from arthritis and experimental colitis. These immunoregulatory strategies prevent the immune response from expelling *D. caninum* from the gastrointestinal tract [25].

Nanotechnology advances affect neurology and have the potential for AD detection [26]. Moreover, nanoparticles (NPs) may greatly favor circulating forms of Aβ, which could yield a “sink effect” and progress AD conditions. It is crucial to use properly designed nanostructures made of biocompatible materials to prevent the negative effects of nanoparticles in AD [26,27]. For instance, superparamagnetic iron oxide nanoparticles (SPIONs) [28] with specific surface coatings are beneficial for both diagnosing and treating AD. We plan to explore various magnetic nanoparticles and their present application in AD, with a particular emphasis on SPIONs. Furthermore, the potential use of *D. caninum* adult worm by-products (ESP) in treating AD remains unexplored in this context. Therefore, the research hypothesis suggests that combination therapy is more effective than monotherapy in the management of AD.

## Materials and methods

### Ethical approval

All particular animal procedures were revised and approved by the state ethics commission and the ethics committee of Alexandria University, Egypt (serial number 0306556 at 7/3/2024; FWA no. 00018699; and IRB no. 00012098).

### Study design

The current study used a true experimental trial design with multiple experimental groups and two control groups.

### Study setting

The present study was conducted at an animal house registered under number 584813328 in Alexandria Ministry of Supply and Internal Trade animal facility, located at coordinates (29.9458 E, 31.1687 N), in conjunction with Pharos University, National Institute of Oceanography and Fisheries (NIOF), and the Faculty of Medicine at Alexandria University, specifically at the department of clinical pharmacology and physiology.

### Sample size

We calculated the sample size using G. power and used the mean value of SOD (mU/mg protein) to distinguish between different brain tissues, such as the cortex and hippocampus. The mean value for the normal group was 14.3609 ± 2.398886, and the mean value for the Alzheimer’s disease group was 8.1802 ± 2.478316. We employed a two-tailed independent t-test to compare the means, using an effect size of 2.534191, an alpha error of 0.05, a power of 0.95, and an allocation ratio of 1:1. The minimum number of rats in each group was six.

### Inclusive criteria of rats

Male albino rats aged 5–6 months, weighing 200–250 grams, had normal biochemical and cognitive parameters.

### Exclusive criteria of rats

Rats’ biochemical and cognitive parameters were not normal related to Alzheimer’s disease.

### Experimental animals cared

I **Experimental rats and husbandry**

The Faculty of Medicine at Alexandria University’s animal house provided 50 male albino rats for the current study. We then cared for the rats in cages, providing them with standard lighting, temperature controls, and unlimited food and water. All research adhered to the recommendations of the Laboratory Animal Care and Ethical Committee (Faculty of Medicine, Alexandria University) and international animal care standards.

II **The technique for scarification and anesthesia to separate the hippocampus and brain cortex tissues of experimental rats**

The combination of ketamine and xylazine (K-X) was used to anesthetize 50 male albino rats. Ketamine is a glutamate n-methyl-D-aspartate antagonist that, when combined with xylazine, produces appropriate anesthesia and analgesia with excellent safety. A α2-adrenergic agonist, xylazine has sedative, muscle-relaxant, and analgesic effects. The intraperitoneal administration of the K-X combination gives rats adequate time for surgical anesthesia and effectively reduces their pain. The lower quadrant of the animal’s abdomen received an anesthetic dose of 100 mg/kg for ketamine and 10 mg/kg for xylazine [29].

To scarify the rats, the abdominal wall was opened, the abdominal skin just inferior to the sternum’s xiphoid process was isolated using forceps, the skin was raised straight up, and the superficial skin was cut off with scissors, revealing the superior and inferior portions of the peritoneum and the peritoneal cavity. The xiphoid process was then lifted, and lateral incisions were made along the subcostal margins just below the process. The front part of the ribcage was then removed from the skull. Finally, the heart was cut away from the anterior wall of the chest using blunt dissection, and the aorta was used to draw blood. The dissection continued into the thoracic cavity, passing through the diaphragm, while ensuring not to lacerate the heart or any major blood vessels [30]. A number 15 scalpel was used to cut the midline from the occipital region to the frontal region, separating the hippocampal tissues by surgical dissection through the skin and the subcutaneous tissue of the skull. Then, we separated the skin, subcutaneous tissue, and periosteum from the osseous tissue laterally and anteriorly using surgical forceps and dissecting scissors. The sliced tissues were turned over from the nasal bone and both orbits’ frontal regions using forceps. Anatomically, the osseous tissues were revealed. Anatomically, we recognized and revealed the sagittal and lambdoid crests, as well as the frontal, parietal, interparietal, and occipital bones [31]. Using iris scissors, the osseous tissue was cut into parallel lines in the caudocranial direction, extending bilaterally along the caudolateral border of the interparietal bone, which also forms the caudal part of the sagittal crest, from the interparietal bone to the frontal bone while maintaining the cerebral tissue. To avoid harming the meningeal or brain tissue, careful procedures were followed [31]. By trying to follow the arbitrary line that went in the middle between the superior borders of both orbital chambers, the frontal bone was cut anteriorly in the cranium. We removed the frontal, parietal, and interparietal bone structures that protected the dorsal surface of the cerebral tissue, revealing the olfactory bulb. The bone tissues that surrounded the dorsal surface of the brain tissue were removed because they reflected off in the form of a cap. Since the meningeal structures that cover and extend between the brain tissue and the cranium serve as protective barriers, care was taken to prevent harm to these structures to minimize damage to the cerebral tissue during dissection [31]. The meninges encircling the cerebral cortex were meticulously removed to separate the brain tissue. The brain tissue was then dissected anteriorly toward the bases of the frontal bone and laterally toward the bases of the temporal bone. To protect the cortical surfaces, small dissectors and curved narrow-pattern forceps were used to cut the brain tissue on both sides at the bases of the temporal bones laterally and the bases of the frontal bones anteriorly [31].

III **Experimental dog model**

**Fig 1:**
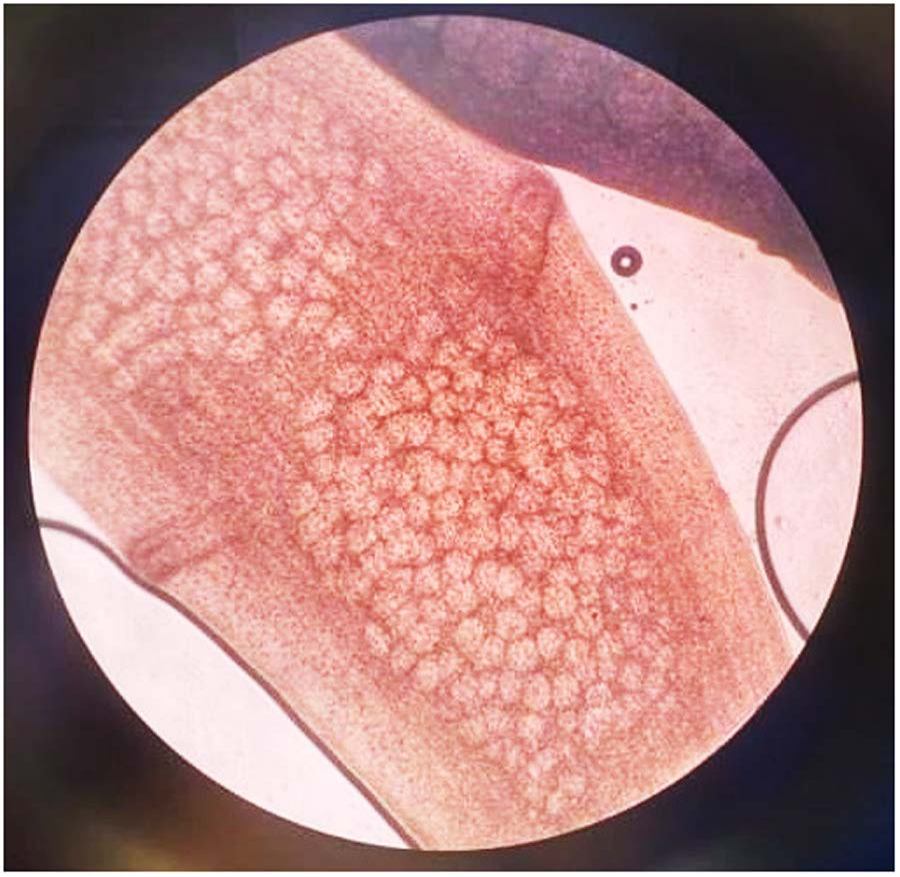
Microscopic investigation of *D. caninum* adult worm showing mature segment, longer than the border, with double sets of genital organs, containing egg capsules.

We randomly selected three stray dogs from the west and center of the Egyptian governorate of Alexandria then cared according to the ethics committee of Alexandria University, Egypt in the animal house registered under number 584813328 in Alexandria Ministry of Supply and Internal Trade. We dewormed the dogs with praziquantel 40 mg/kg and then administered a purgative. We collected *D. caninum* adult worms from dog guts at Pharos University, washed them with regular saline solution, and then transmitted them to the Drug Research Center at the Faculty of Pharmacy in Alexandria, Egypt, for analysis and categorization. We examined proglottids macroscopically after they recovered, washed them in body-warm normal saline, and observed mature segments microscopically (**Fig 1**) [32].

### Drug intervention preparation

I **Colchicine and artificial cerebrospinal fluid (ACSF)**

The supplier of colchicine was Sigma Aldrich Pharmaceutical Co. Freshly generated artificial cerebrospinal fluid (ACSF) had the following components in milliliters: sodium chloride (NaCl) 147, potassium chloride (KCl) 2.9, magnesium chloride (MgCl₂) 1.6, calcium chloride (CaCl₂) 1.7, and dextrose 2.2 [33].

II **Superparamagnetic iron oxide nanoparticles (SPIONs): biosynthesis and characterization**

SPIONs were produced via a biological process. In a conventional synthesis, 0.001 M of ferrous sulphate (FeSO_4_.7H_2_O) and ferric chloride (FeCl₃) substrates were combined with one gram of *Bacillus velezensis* SMR cells. The mixtures were incubated at 37°C while being agitated at 150 rpm. Throughout the 24-hour incubation period, the nanoparticles were permitted to grow. Visual observation was made possible by the biotransformed solutions’ hue shift to brown or black. The produced SPIONs were rinsed threefold with distilled water and purified using an external magnet in order to get them ready for more research [34].

For a better understanding of the global properties of the created SPIONs, a range of characterization techniques have been applied. These contain high-resolution transmission electron microscope (TEM) with the JEM-2100UHR, energy-dispersive X-ray (EDX) examination with a scanning electron microscope (SEM) Jeol JSM-5300 equipped with an EDX unit, and X-ray diffraction (XRD) with a Bruker XRD-D2 Phaser. The functionalization groups were verified by Fourier transform infrared spectroscopy (FTIR) using a Bruker Tensor 37, and the magnetic properties were assessed using a Lakeshore 7410 vibrating sample magnetometer (VSM). Using these techniques, we can examine the crystal phase construction, surface morphology, pattern of sizes, chemical constitution, dimensional arrangement of the functional groups, and magnetism of the generated SPIONs [34].

III **Preparation of the excretory-secretory product (ESP)**

To minimize contamination, we sterilized the gut microbiota of adult *Dipylidium caninum* worms three times in phosphate-buffered saline (PBS) holding 1% formaldehyde. We froze the worms whole using liquid nitrogen and pulverized them into powder [35]. We cultivated them in PBS (2% glucose) with 1% human plasma and cultured them for a full day at 37°C. We precipitated the dense filtrate overnight at -20°C using acetone (5 Vols) and 20% trichloroacetic acid. We collect the precipitates at 10,000 rpm for 10 minutes at 4°C using a cold centrifuge. We gather the pellet, freeze-dry it, immerse it in lysate buffer, and ultrasound-treat it (with ice) until the solution turns translucent. We centrifuged the homogenate for 15 minutes at 4°C and 12,000 rpm. The supernatant contains ESPs [36]. We administer seven intraperitoneal 0.75% (0.3 ml/100 g) injections on alternative days so extended to 14 days [37].

IV **The** **combination** **between** **Superparamagnetic** **iron oxide** **nanoparticles** **(SPIONs)** **and** ***Dipylidium** **caninum*** **adult** **worms**

The entire worms were transferred to a standard saline solution enriched with SPIONs mixture (Fe_3_O_4_-SPIONs and Fe_2_O_3_-SPIONs) at different concentrations of 50, 200, and 400μg/ml while maintaining aseptic conditions. The concentrations were prepared via an immediate-use combination of liquid paraffin and Tween 80 (v/v). The whole worms were then cultured for 24 hours at 37°C. For a day, the solvent control worms were cultivated in a standard saline solution that contained 0.2% (v/v) liquid paraffin and Tween 80. Immediately following the first washing, the standard control worms were repaired. SEM was used to evaluate the effect of SPIONs on worms following a 24-hour incubation period [38].

V **Cortical and hippocampal tissues preparation**

Following cleaning, we homogenized the extracted cortical and hippocampal tissues 1:9 in phosphate-buffered saline (pH 7.4). After centrifuging the homogenate at 4°C, we immediately used the supernatants to estimate several biochemical indicators.

### The intervention processed for intracerebroventricular administration of colchicine to induce Alzheimer’s disease for 40 rats

**Fig 2:**
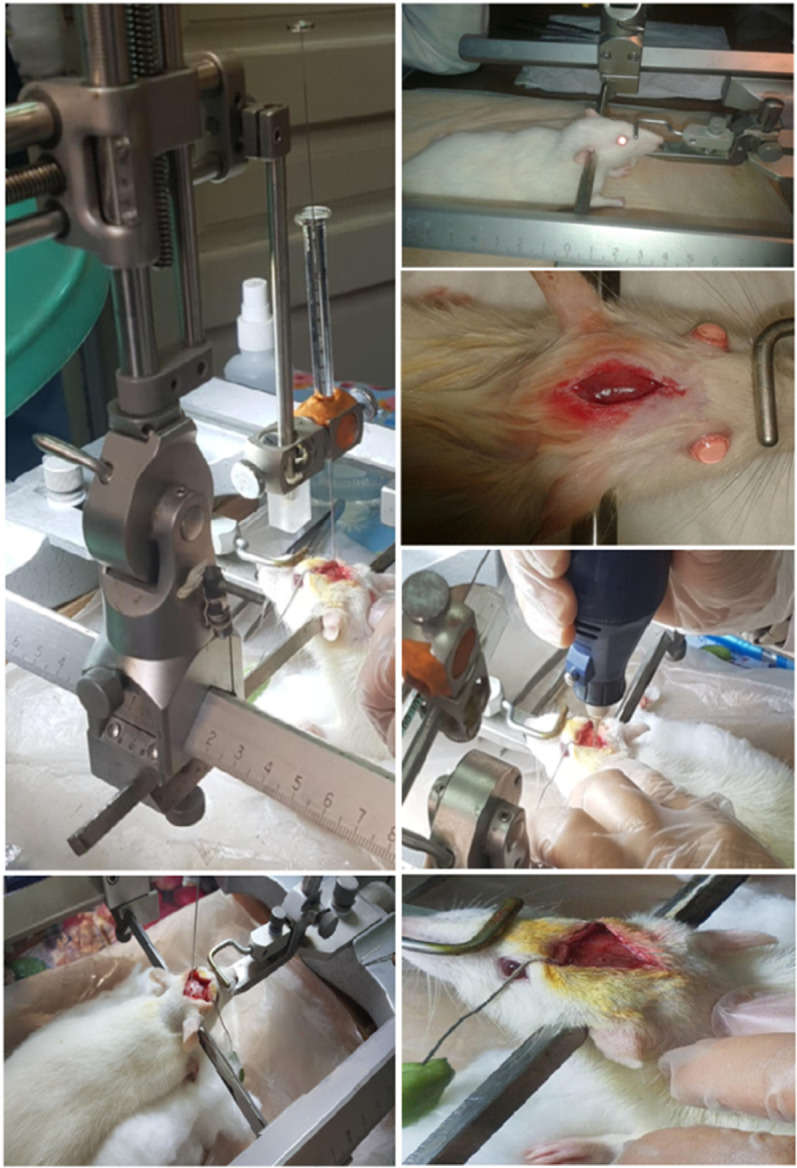
Surgery and intracerebroventricular administration of colchicine by a Hamilton microsyringe.

Under strict aseptic circumstances, animals were inserted in a stereotaxic instrument and administered an intraperitoneal (i.p.) 45 mg/kg thiopental sodium anesthetic. Using a stereotaxic device (Kopf, Germany), surgery was performed in compliance with the previously described protocol [39]. The scalp was cut midline sagittal, the pericranial muscles and fascia were retracted laterally, and the head was placed inside a frame. Using a drill fitted with a minute dental bur, two cavities were created bilaterally in the skull at the stereotaxic coordinates of 0.8 mm posterior to the bregma, 1.8 mm lateral to the sagittal suture, and 3.6 mm below the cortical surface (Paxinos and Watson atlas) [33,40]. A 28-gauge Hamilton® microsyringe (10 μL) was used to inject 15 μg of colchicine diluted in 10 μL of ACSF bilaterally into the lateral ventricles. The microsyringe was maintained *in situ* for two minutes after the injection rate of two microliters per minute in order to stop the injected fluid from being removed. The scalp was then sealed with a suture. Rats that received ACSF injections as controls were used to test the ICV ACSF injection technique. Rats were given gentamicin (5 mg/kg, i.p.) after surgery to avoid sepsis and were monitored closely until they began to move around on their own [33] (**Fig 2**).

### Experimental design

We randomly assigned 50 rats into 5 main groups, with each group contained 10 rats:

**Group I** was negative control which kept in standard care regulation according to ethics committee of Alexandria University, Egypt.

**Group II** was the positive control (colchicine-induced AD group). They gave each rat a single ICV injection of 15 µg of colchicine mixed with ACSF (7.5 µg in 5 µL/site), which was done bilaterally into the lateral ventricles using a Hamilton microsyringe. The next day after surgery, rats got 1 mL of 2% gum acacia orally daily for 25 days.

**Group III** was the intervention-positive group that was given *D. caninum* ESP and one dose of colchicine (15 µg/rat, i.c. injection). The next day after surgery, rats received *D. caninum* ESP [0.75% (0.3 ml/100 g)] intraperitoneal 7 times on for 14 alternating days.

**Group IV** was the intervention-positive group treated with Nano-SPIONs; typically a single dose of colchicine (15 μg/rat, ICV injection). The next day after surgery, rats received Nano-SPIONs embedded in 2% gum acacia (50 mg/kg/day, oral gavage) for 25 days.

**Group V** was the intervention-positive group, received a combination of Nano-SPIONs and *D. caninum* ESP, along with a single dose of colchicine (15 g/rat, icv injection). The day after surgery, the rats were given *D. caninum* ESP [0.75% (0.3 ml/100 g)] intraperitoneal seven times, they were also given Nano-SPIONs embedded in 2% gum acacia (50 mg/kg/day, orally), for 25 days.

### Intervention drugs assessment

I **For biochemical parameters**

Four biochemical parameters were measured: malondialdehyde (MDA), superoxide dismutase (SOD) activity, total protein, and amyloid beta and nuclear factor-kappa B. Immediately after sacrifice, we preserved the tissues in -80 and sent them for biochemical analyses.

1. **Determination of MDA:** We measured the MDA in the homogenate of rats’ cortical and hippocampal tissues using Draper and Hadley’s colorimetric technique [42].
2. **Determination of SOD activity:** We measured the activity of SOD using a spectrophotometric test, the pyrogallol technique of Marklund and Marklund [43].
3. **Determination of total protein:** We employed a modified version of the Lowry et al. method [44] to determine the amount of protein in each sample. It is believed that a molecule that was created by the reaction between the alkaline copper phenol reagent and tryptophan and tyrosine residues in the protein sample was what gave the colour. We constructed a standard curve with cow’s albumin to determine the protein content of each sample.

II **For** **cognitive** **parameters**

#### Morris water maze task (MWM).

We investigated the formation and maintenance of spatial navigation memory using a MWM task. The animals learned to swim 180 centimeters in a 60-centimeter-high circular pool. We flooded the pool to a depth of 40 cm, ensuring the water temperature was between 22 and 2°C. We connected a 9-cm-diameter white circular platform to a vertical support and positioned it 2 cm below the water’s surface. We incorporated milk powder into the water to make the platform undetectable. The north, south, east, and west beginning points on the wall separated the pool into four quadrants. Throughout the experiment, only the platform in one of the four quadrants could exit the water. We hung a colorful flag outside the pool to help the rat find the submerged platform [40].

### Hidden platform test (testing for retention of learned tasks)

Starting on day 18, we subjected the rats to this test for three days straight, giving them four trials per training session. Starting positions varied for all four trials. One of the beginning locations initiated a trial by releasing the rat into the maze facing the pool wall. The maximum time to discover an escape platform was two minutes, or 120 seconds. If a rat failed to find its way onto the platform within that timeframe, we reopened the experiment. After that, we positioned it and held it there for fifteen seconds. We placed the rat in a cage next to the maze after each trial, giving it ten seconds to relax before initiating the next one. Once the fourth trial is concluded, we carefully dried the rat with a towel before returning it to its cage. We thoroughly mixed the water between the two rat sessions to prevent any potential scent traces. We assessed the drug intervention using seven cognitive parameters over 21 days.

1. **Initial acquisition latency (IAL):** The first cognitive parameter was the average time a rat takes to reach the platform on day one.
2. **The rats’ escape retention latencies (RLs), which were estimated twice (first and second):** which were made up of the first and second RLs, the second cognitive parameter. The rats could remember the task they had learned on the second and third days.
3. **The probe (retrieval) trial**: The third cognitive parameter tested the animal’s ability to recall its spatial memory. We removed the platform from the pool on day 21, 24 hours after the previous maze retention test, and gave the taught animal 60 seconds to swim at its own pace. We used the duration of the rat’s stay in the target quadrant, where the platform was during the acquisition assessment, to evaluate the spatial accuracy of the probe session.
4. **Passive avoidance (PA) task which was estimated twice (initial and retention):** the fourth cognitive parameter was included, PA effect initial latency and PA effect step retention latency, which during at day 21, the step-through passive avoidance test was employed to evaluate the unpleasant memory by looking at memory and training in response to a stressful stimulus. The two similar chambers (22 × 21 × 22 cm each) that comprised the training equipment were connected by a guillotine door measuring 5 × 22 cm. There was light in one compartment and darkness in the other. The stainless steel shock grid comprised the chamber’s floor. Before the experiment, we gently placed each rat individually in the illuminated compartment with their backs facing the guillotine door, which opened after five seconds. We allowed the rats to investigate the device for sixty seconds. We repeated the process after fifteen minutes, using the time it took the rat to enter the dark chamber to determine its initial step-through latency (ISTL) for the acquisition session. We prohibited rats with an ISTL greater than 300 seconds from participating in future trials. When the door closed, the tested rat was fully inside the dimly illuminated chamber. We restored the tested rat to its cage after administering a three-second electric foot shock (75V, 0.2 mA, 50 Hz). We cleaned both compartments between training sessions to prevent scent confusion. We measured the step-through retention latency (STRL) up to 300 seconds after 24 hours without foot shock, using the same method as the acquisition experiment. If the animal never entered the dark room, we recorded a maximum delay of three hundred seconds. This task teaches the animal to avoid places where they have experienced trauma. A reduction in the STRL time during this exercise indicates memory impairment [41].
5. **Locomotor activity in rats:** We assessed behavioral phenotypes, circadian rhythm, and brain arousal using locomotor activity, the movement behavior that rodents display, without needing learning or training.

### 4. The Determination of Amyloid Beta, and Nuclear Factor-Kappa B

Followed the manufacturer’s instructions (Chongqing Biospes Co., Ltd., China), quantitative measurements of amyloid beta (Aβ1–42) and nuclear factor-kappa B (NF-κB) were performed in hippocampal and cortical homogenates using rat-specific ELISA kits. Both the NF-κB (BYEK1184) and Aβ1–42 (BYEK2988) rat ELISA kits are available.

### Statistical analysis

The data were collected in a spreadsheet in Excel, and then, by using the SPSS program version 20, firstly, the test of normal distribution by the Shapiro-Wilk test was done for quantitative variables, which were represented by mean ± SD. At a significance level of 5%, discriminant analysis was used to find the important biochemical differences between the brain cortex and hippocampus in AD. This was followed by a MANOVA test that compared all biochemical and cognitive parameters as dependent variables to the 5 study groups as independent variables. Finally, the Post Hoc test (Bonferroni correction) was used to account for multiple pairwise comparisons between the intervention study groups and the negative control group (references group). Also we can calculated the multivariate Test Wilks’ Lambda was the most suitable due to small sample size to evaluate the total efficacy of intervention program and also tests of between-subjects effects to predicate the effect size of intervention program on each all biochemical and cognitive parameters by using Partial Eta Squared test which its value ≥ 0.1 indicated large effect size, but the values ranged from 0.02 to 0.1 revealed medium effect size and the value ≤ 0.01 designated small effect size, and the Adjusted R Squared related to the variation in dependent variables (biochemical and cognitive parameters) depended on the independent variables (intervention programs) by the values of Adjusted R Squared after multiply by 100 to change the Adjusted R Squared to percentage. Finally, we used the Log Rank (Mantel-Cox) Kaplan-Meier curve to figure out how the 21-day study period affected changes in cognitive parameters based on independent variables (intervention programs). The shortest matched time at the X-axis and the median point at 0.5 on the Y-axis showed the faster intervention drug. We then used the AMOS program to figure out the correlation r value and regression beta (B-slope) coefficient for each cognitive parameter to show how they were related to each other and how much they improved cognitive behaviors.

## Results

**Fig 3:**
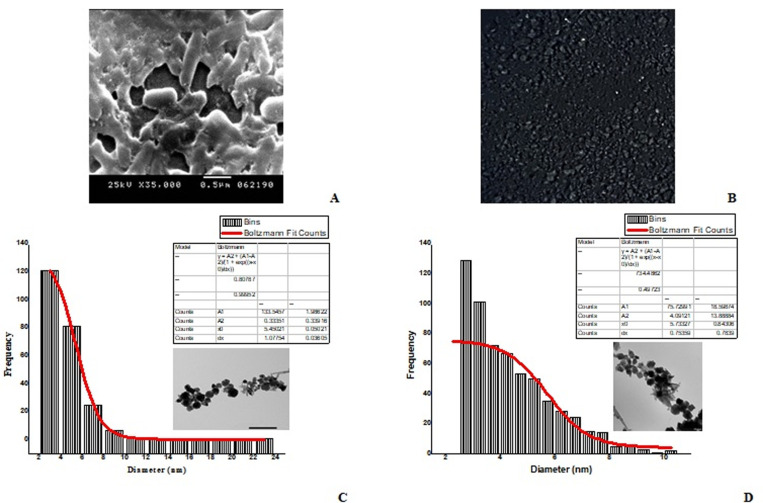
*Bacillus velezensis* SMR cells (A) produced a mixture of SPIONs (Fe_3_O_4_ and Fe_2_O_3_) as black powders **(B).** **The** **size** **distribution** **curve** **for** **Fe_3_O_4_** **and** **Fe_2_O_3_** **showed** **that** **they** **were** **mostly** **5** **and** **6** **nm** **in size** **(C and D),** **respectively.**

We cultured *B. velezensis* SMR cells (Fig 3A) with iron substrates for 24 hours. After adding salt solutions, the bacterial-substrate suspensions showed a color shift from colorless to black, confirming the synthesis of SPIONs as black powders. After purifying the dried black powders (Fig 3B) using an external magnet, we analyzed them for characterization. Transmission electron microscope (TEM) revealed that the nanoparticles varied in size with irregular spherical shape. The average diameter of the Fe_3_O_4_ powder was 5 nm (Fig 3C**),** and that of the Fe_2_O_3_ powder was 6 nm (Fig 3D**).**

The biosynthesized SPIONs have an FT-IR spectrum that shows O-H groups on the surface, Fe-O stretching vibrations for both types of SPIONs, and C-O stretching vibrations for Fe_3_O_4_ and C = O for Fe_2_O_3_ (Table 1**).** These peaks indicate the presence of biomolecules, stabilizing the SPIONs with surface biomolecules such as peptides. They act as capping agents. The EDX analysis revealed that the biosynthesized SPIONs primarily consist of 100% Fe and O, with a chemical composition of Fe_3_O_4_ (Fe: 65.93–O: 34.07) and Fe_2_O_3_ (Fe: 61.65–O: 38.35) (Table 1**).** The study indicates that SPIONs have distinct magnetic responses at the nanoscale. We studied the magnetic properties of these nanoparticles using VSM. They had closed hysteresis loops with Ms values of 54.17 and 52.09 emu/g for Fe_3_O_4_ and Fe_2_O_3_, low coercivity values of 59.253 and 75.03 Oe, and retentivity values of 6.450 and 6.001 emu/g. This suggests that they are extremely superparamagnetic (Table 1**).** The study examined the structural and crystalline nature of synthesized SPIONs using X-ray diffraction analysis. Both materials had a cubic spinel structure. There was a main diffraction peak at 35.07° for Fe_3_O_4_ and 35.01° for Fe_2_O_3_ (Table 1). These peaks correspond to the lattice plane (hkl) < 311 > . We mixed the two types of SPIONs (Fe_3_O_4_-SPIONs and Fe_2_O_3_-SPIONs) and tested their effects on the adult worm *D. caninum*.

**Table 1:** The biosynthesis and characterization of superparamagnetic iron oxide nanoparticles (SPIONs).

Microbial factory	Accession number	Approach	Iron substrate	SPIONs type	Size (nm)	Functional groups	Purity	Magnetic properties	Characteristic peak and phase structure
*Bacillus velezensis* SMR	MT232963	Bottom up (Biological)	FeCl_3_	Fe_3_O_4_	5	O-H C-O Fe-O	100% (Fe:65.93 –O:34.07)	Ms: 54.17 emu/g Hci: 59.253 Oe Mr: 6.450 emu/g	35.07° ≈ < 311> Cubic spinel
			FeSO_4_	Fe_2_O_3_	6	O-H C = O Fe-O	100% (Fe:61.65 –O:38.35)	Ms: 52.09 emu/g Hci:75.03 Oe Mr: 6.001 emu/g	35.01° ≈ < 311> Cubic spinel

Ms: Saturation Magnetization, Hci: Coercivity, Mr: Retentivity

**Fig 4:**
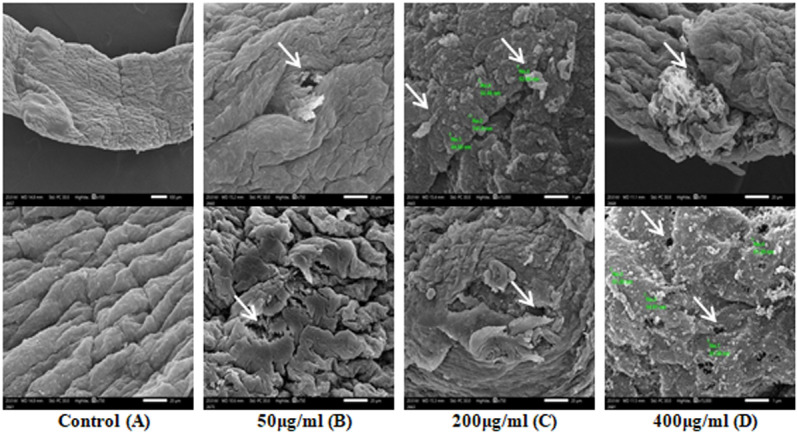
Displays the SEMs of an adult *D. caninum.* **The image displays a typical control cestode, incubated in a normal saline solution (0.2% (v/v) mixture of liquid paraffin and Tween 80**) **(A).** **The** **SEM images (B, C, and D) depict the skin following a 24-hour.**

Compared to the control specimens, the changes in mature cestodes mostly affected the tegument body surface in almost all specimens studied (Fig 4A). After being incubated *in vitro* with 50, 200, or 400 μg/ml SPIONs mixture for 24 hours, most of the specimens had severely tucked and distorted epidermis (Fig 4B). Increasing the dose of the SPIONs mixture results in significant distortion of the tegument surface, indicating increased skin damage. Several white lesions appeared (Fig 4C), including lacerations and the appearance of surface pores (Fig 4D).

The results in **Table 2** showed that the overall cross-validated grouped cases were correctly classified based on current biochemical parameters at 92.0%, which was higher than the cutoff value of 62.5%. The first parameter was SOD (mU/mg protein), which was thought to be the best way to diagnose Alzheimer’s disease between the brain cortex and hippocampus because it had the highest F value (10.740%, P = 0.002), and the second was AB1–42 (ng/mg protein) (F = 10.444, P = 0.002). Conversely, tests of equality of group means revealed that the other biochemical parameters, NF-kB (pg/mg protein) and MDA (nmol/g tissue), did not significantly differ between the brain cortex and hippocampus in Alzheimer’s disease.

**Table 2:** Demonstrates the discrimination analysis of biochemical parameters in different brain tissues.

Tissue type	Biochemical parameters	Mean± SD	Tests of Equality of Group Means			Cross-validated grouped cases correctly classified
			Biochemical parameters	F	Sig.	
Brain Cortex	AB1-42 (ng/mg protein)	7.75±2.63	AB1-42 (ng/mg protein)	10.444	0.002	92.0%
	NF-kB (pg/mg protein)	43.07±15.81				
	MDA (nmol/g Tissue)	1.63±0.63	NF-kB (pg/mg protein)	3.020	0.089	
	SOD (mU/mg protein)	12.66±2.91				
Hippocampus	AB1-42 (ng/mg protein)	5.44±2.403	MDA (nmol/g Tissue)	0.622	0.434	
	NF-kB (pg/mg protein)	34.81±17.72				
	MDA (nmol/g Tissue)	1.47±0.79	SOD (mU/mg protein)	10.740	0.002	
	SOD (mU/mg protein)	9.79±3.25				

Discrimination analysis at Significant at the.05 level

**Table 3** reveals that we evaluated all tested drug groups based on changes in biochemical parameters. The multivariate test Wilks’ lambda showed that all programs had a significantly large effect size on changing Alzheimer’s disease (P = 0.000, Partial Eta Squared = 0.380). The tests of the effect of the drugs on different biochemical parameters also showed that all of the drugs had a significantly large effect size for each biochemical parameter (P values ≤ 0.05 and all Partial Eta Squared values above 0.1). Furthermore, the adjusted R-squared showed that NF-kB (pg/mg protein) had the highest value of 0.705, which means that all intervention drugs caused a 70.5% change in NF-kB (pg/mg protein). MDA (nmol/g tissue) came in second, with an adjusted R-squared of 0.645.

**Table 3:** Shows the biochemical parameters changes according to intervention study groups.

Biochemical test	Study groups	Mean± SD	Multivariate Test Wilks' Lambda		Tests of Between-Subjects Effects		Adjusted R Squared
			Sig.	Partial Eta Squared	Sig.	Partial Eta Squared	
AB1-42 (ng/mg protein)	Negative control	3.4694±1.135923	0.000	0.380	0.000	0.551	0.512
	Positive control (Colchicine induced AD group)	8.7032±3.089067					
	Intervention positive group treated with *D. caninum* ESP)	8.9168±1.386712					
	Intervention positive group treated with Nano-SPIONs)	6.1663±2.099578					
	Intervention positive group treated with combination of both Nano-SPIONs and *D. caninum* ESP	5.7553±1.163621					
NF-kB (pg/mg protein)	Negative control	12.6985±4.508373			0.000	0.729	0.705
	Positive control (Colchicine induced AD group)	50.7697±16.435638					
	Intervention positive group treated with *D. caninum* ESP)	53.5091±4.063977					
	Intervention positive group treated with Nano-SPIONs)	41.0036±9.589005					
	Intervention positive group treated with combination of both Nano-SPIONs and *D. caninum* ESP	36.7495±5.825813					
MDA (nmol/g Tissue)	Negative control	0.6686±0.088177			0.000	0.674	0.645
	Positive control (Colchicine induced AD group)	2.3082±0.638826					
	Intervention positive group treated with *D. caninum* ESP)	2.0739±0.405792					
	Intervention positive group treated with Nano-SPIONs)	1.4132±0.503551					
	Intervention positive group treated with combination of both Nano-SPIONs and *D. caninum* ESP	1.3124±0.281845					
SOD (mU/mg protein)	Negative control	14.3609±2.398886			0.000	0.445	0.395
	Positive control (Colchicine induced AD group)	8.1802±2.478316					
	Intervention positive group treated with *D. caninum* ESP)	9.2757±2.936761					
	Intervention positive group treated with Nano-SPIONs)	12.5579±3.232117					
	Intervention positive group treated with combination of both Nano-SPIONs and *D. caninum* ESP	11.7694±1.916576					

MANOVA test at significant at the.05 level

**Table 4** showed that, in relative to the pairwise comparison between the all intervention drugs and negative control as reference group for modulation of Alzheimer’s disease according to all biochemical parameters said that; the least means difference between the negative control as a reference group and the intervention drug groups for AB1–42 (ng/mg protein), NF-kB (pg/mg protein), and MDA (nmol/g Tissue) (-2.286, -24.051, and -0.644, respectively) were related to group V (the intervention positive group treated with a combination of both Nano-SPIONs and *D. caninum* ESP), except with regard to SOD (mU/mg protein) the least mean difference was1.803 belong to intervention positive group treated with Nano-SPIONs) so we can conclude that the curative intervention drug for biochemical parameters modulation of Alzheimer’s disease was group V.

**Table 4:** Displays the pairwise comparisons of biochemical parameters among different study groups compared with negative control group (reference group).

Dependent Variable	Treatment Groups (I)	Treatment Groups (J)	Mean Difference (I-J)	Sig.	95% Confidence Interval for Difference	
					Lower Bound	Upper Bound
AB1–42 (ng/mg protein)	Negative control	Positive control (Colchicine induced AD group)	-5.234	0.000	-7.775-	-2.693-
		Intervention positive group treated with *D. caninum* ESP)	-5.447	0.000	-7.988-	-2.907-
		Intervention positive group treated with Nano-SPIONs)	-2.697	0.030	-5.238-	-.156-
		Intervention positive group treated with combination of both Nano-SPIONs and *D. caninum* ESP	-2.286	0.109	-4.827-	.255
NF-kB (pg/mg protein)	Negative control	Positive control (Colchicine induced AD group)	-38.071	0.000	-50.355-	-25.787-
		Intervention positive group treated with *D. caninum* ESP)	-40.811	0.000	-53.094-	-28.527-
		Intervention positive group treated with Nano-SPIONs)	-28.305	0.000	-40.589-	-16.021-
		Intervention positive group treated with combination of both Nano-SPIONs and *D. caninum* ESP	-24.051	0.000	-36.335-	-11.767-
MDA (nmol/g Tissue)	Negative control	Positive control (Colchicine induced AD group)	-1.640	0.000	-2.204-	-1.075-
		Intervention positive group treated with *D. caninum* ESP)	-1.405	0.000	-1.970-	-.841-
		Intervention positive group treated with Nano-SPIONs)	-0.745	0.003	-1.309-	-.180-
		Intervention positive group treated with combination of both Nano-SPIONs and *D. caninum* ESP	-0.644	0.016	-1.208-	-.079-
SOD (mU/mg protein)	Negative control	Positive control (Colchicine induced AD group)	6.181	0.000	2.706	9.656
		Intervention positive group treated with *D. caninum* ESP)	5.085	0.001	1.610	8.560
		Intervention positive group treated with Nano-SPIONs)	1.803	1.000	-1.672-	5.278
		Intervention positive group treated with combination of both Nano-SPIONs and *D. caninum* ESP	2.591	0.329	-.883-	6.066

Based on estimated marginal means, the mean difference was significant at the.05 level. Adjustment for multiple comparisons: Bonferroni

**Table 5** demonstrates the evaluation of all tested drug groups based on changes in the cognitive parameters of MWM. The multivariate test, Wilks’ lambda, showed that all of the drug groups had a significant effect on Alzheimer’s disease, with an effect size of 0.000 (P = 0.000) and a partial eta squared value of 0.750. The tests of the differences between cognitive parameters of MWM also showed a significant effect, with an effect size of all P values ≤ 0.05 and all Partial Eta Squared values above 0.1 for each cognitive parameter of MWM. Furthermore, the adjusted R-squared values for all cognitive parameters of MWM were above 0.9, indicating that the variability in these parameters across all intervention drug groups was above 90%.

**Table 5:** Shows the cognitive parameters of MWM changes according to intervention study groups.

Cognitive Parameters	Study Groups	Mean± SD	Multivariate Test Wilks' Lambda		Tests of Between-Subjects Effects		Adjusted R Squared
			Sig.	Partial Eta Squared	Sig.	Partial Eta Squared	
AL initial	Negative control	47.625±3.2064	0.000	0.750	0.000	0.953	0.948
	Positive control (Colchicine induced AD group)	92.725±1.4503					
	Intervention positive group treated with *D. caninum* ESP)	92.025±0.7587					
	Intervention positive group treated with Nano-SPIONs)	46.125±1.6552					
	Intervention positive group treated with combination of both Nano-SPIONs and *D. caninum* ESP	66.225±9.9565					
RLs.1st	Negative control	28.400±2.7467			0.000	0.971	0.968
	Positive control (Colchicine induced AD group)	80.700±3.1881					
	Intervention positive group treated with *D. caninum* ESP)	87.675±2.2823					
	Intervention positive group treated with Nano-SPIONs)	26.925±1.6118					
	Intervention positive group treated with combination of both Nano-SPIONs and *D. caninum* ESP	51.70±9.1445					
RLs.2nd	Negative control	23.25±2.1890			0.000	0.956	0.952
	Positive control (Colchicine induced AD group)	77.20±2.1370					
	Intervention positive group treated with *D. caninum* ESP)	84.60±3.1780					
	Intervention positive group treated with Nano-SPIONs)	20.625±1.4398					
	Intervention positive group treated with combination of both Nano-SPIONs and *D. caninum* ESP	40.100±12.7520					
Probe	Negative control	45.70±0.949			0.000	0.946	0.941
	Positive control (Colchicine induced AD group)	22.00±3.712					
	Intervention positive group treated with *D. caninum* ESP)	16.10±1.595					
	Intervention positive group treated with Nano-SPIONs)	46.20±2.394					
	Intervention positive group treated with combination of both Nano-SPIONs and *D. caninum* ESP	36.20±4.962					

MANOVA test at significant at the.05 level

**Table 6** in qualified to the pairwise comparison between the all-intervention-drug group and negative control as the reference group for modulation of Alzheimer’s disease according to all cognitive parameters of MWM supposed that the least means difference between negative control as the reference group and intervention drug groups for AL initial, RLs.1st, RLs.2nd, and Probe (1.500, 1.475, 2.625, and -0.500, respectively) with insignificant differences when compared with the negative control group were related to group IV (intervention positive group treated with Nano-SPIONs), so we can conclude that the curative intervention drug for cognitive parameters of MWM modulation of Alzheimer’s disease was group IV.

**Table 6:** Presents the pairwise comparisons of cognitive parameters of MWM among different study groups compared with negative control group (reference group).

Dependent Variable	Study Groups (I)	Study Groups (J)	Mean Difference (I-J)	Sig.	95% Confidence Interval for Difference	
**Lower Bound**	**Upper Bound**
AL initial	Negative control	Positive control (Colchicine induced AD group)	-45.100^*^	0.000	-51.427	-38.773
		Intervention positive group treated with *D. caninum* ESP	-44.400^*^	0.000	-50.727	-38.073
		Intervention positive group treated with Nano-SPIONs	1.500	1.000	-4.827	7.827
		Intervention positive group treated with combination of both Nano-SPIONs and *D. caninum* ESP	-18.600^*^	.000	-24.927	-12.273
RLs.1^st^	Negative control	Positive control (Colchicine induced AD group)	-52.300	.000	-58.468	-46.132
		Intervention positive group treated with *D. caninum* ESP	-59.275^*^	0.000	-65.443	-53.107
		Intervention positive group treated with Nano-SPIONs	1.475	1.000	-4.693	7.643
		Intervention positive group treated with combination of both Nano-SPIONs and *D. caninum* ESP	-23.300	.000	-29.468	-17.132
RLs.2^nd^	Negative control	Positive control (Colchicine induced AD group)	-53.950	0.000	-61.962	-45.938
		Intervention positive group treated with *D. caninum* ESP	-61.350^*^	0.000	-69.362	-53.338
		Intervention positive group treated with Nano-SPIONs	2.625	1.000	-5.387	10.637
		Intervention positive group treated with combination of both Nano-SPIONs and *D. caninum* ESP	-16.850	0.000	-24.862	-8.838
Probe	Negative control	Positive control (Colchicine induced AD group)	23.700	0.000	19.628	27.772
		Intervention positive group treated with *D. caninum* ESP	29.600	0.000	25.528	33.672
		Intervention positive group treated with Nano-SPIONs	**-0.500**	1.000	-4.572	3.572
		Intervention positive group treated with combination of both Nano-SPIONs and *D. caninum* ESP	9.500	0.000	5.428	13.572

Based on estimated marginal means, the mean difference was significant at the.05 level. Adjustment for multiple comparisons: Bonferroni

**Table 7**, indicated that; as admiration to the evaluation of all intervention drug groups according to change in cognitive parameters of PA designated that; according to the multivariate test wilks’ lambda the overall the all intervention drug groups were significant large effect size (P = 0.000, Partial Eta Squared = 0.618) on modulation of Alzheimer’s disease and also tests of between cognitive parameters of PA effect the only PA Effect step retention latency was significant for all intervention drugs group with large effect size (P = 0.000, Partial Eta Squared = 0.936) and highest Adjusted R Squared = 0.931, that means the variability in PA Effect step retention latency related to intervention drugs group by 93.1%, on other hand the PA effect initial latency and locomotor. The effects of the drug groups were insignificant and had a small effect size.

**Table 7:** Shows the cognitive parameters of passive avoidance (PA) changes according to intervention study groups.

Cognitive Parameters	Study Groups	Mean± SD	Multivariate Test Wilks' Lambda		Tests of Between-Subjects Effects		Adjusted R Squared
			Sig.	Partial Eta Squared	Sig.	Partial Eta Squared	
PA Effect initial latency	Negative control	27.10±4.886	0.000	0.618	0.575	0.061	0.022
	Positive control (Colchicine induced AD group)	26.40±5.420					
	Intervention positive group treated with *D. caninum* ESP)	26.20±4.984					
	Intervention positive group treated with Nano-SPIONs)	23.70±3.498					
	Intervention positive group treated with combination of both Nano-SPIONs and *D. caninum* ESP	25.10±5.507					
PA Effect step retention latency	Negative control	298.70±2.111			0.000	0.936	0.931
	Positive control (Colchicine induced AD group)	114.80±4.341					
	Intervention positive group treated with *D. caninum* ESP)	97.80±4.849					
	Intervention positive group treated with Nano-SPIONs)	299.00±1.764					
	Intervention positive group treated with combination of both Nano-SPIONs and *D. caninum* ESP	252.2054.002					
Locomotor. Activity	Negative control	48.601.647			0.935	0.018	0.069
	Positive control (Colchicine induced AD group)	48.402.366					
	Intervention positive group treated with *D. caninum* ESP)	48.702.497					
	Intervention positive group treated with Nano-SPIONs)	48.302.406					
	Intervention positive group treated with combination of both Nano-SPIONs and *D. caninum* ESP	47.901.853					

Based on estimated marginal means at 5% significant level

According to **Table 8**, the pairwise comparison between the all-intervention drug group and the negative control, according to all cognitive parameters of PA, revealed that; the least mean difference in initial latency and locomotor activity was represented in group III by 0.900 and -0.100, respectively. On the other hand, in relation to step retention latency, the least mean difference -0.300, belonged to group IV. So, we can conclude that the curative intervention drug for the cognitive parameters of PA modulation of Alzheimer’s disease was group III, followed by group IV.

**Table 8:** Displays the pairwise comparisons of cognitive parameters of passive avoidance (PA) among different study groups compared with negative control group (reference group).

Dependent Variable	Study Groups (I)	Study Groups (J)	Mean Difference (I-J)	Sig.	95% Confidence Interval for Difference	
**Lower Bound**	**Upper Bound**
PA. Effect. Initial. latency	Negative control	Positive control (Colchicine induced AD group)	0.700	1.000	-5.785-	7.185
		Intervention positive group treated with *D. caninum* ESP	0.900	1.000	-5.585-	7.385
		Intervention positive group treated with Nano-SPIONs	3.400	1.000	-3.085-	9.885
		Intervention positive group treated with combination of both Nano-SPIONs and *D. caninum* ESP	2.000	1.000	-4.485-	8.485
PA. Effect.step. retention.latency	Negative control	Positive control (Colchicine induced AD group)	183.900^*^	0.000	151.745	216.055
		Intervention positive group treated with *D. caninum* ESP	200.900^*^	0.000	168.745	233.055
		Intervention positive group treated with Nano-SPIONs	-0.300	1.000	-32.455-	31.855
		Intervention positive group treated with combination of both Nano-SPIONs and *D. caninum* ESP	46.500^*^	0.001	14.345	78.655
Locomotor. Activity	Negative control	Positive control (Colchicine induced AD group)	0.200	1.000	-2.678-	3.078
		Intervention positive group treated with *D. caninum* ESP	**-0.100**	1.000	-2.978-	2.778
		Intervention positive group treated with Nano-SPIONs	0.300	1.000	-2.578-	3.178
		Intervention positive group treated with combination of both Nano-SPIONs and *D. caninum* ESP	0.700	1.000	-2.178-	3.578

Based on estimated marginal means, the mean difference was significant at the.05 level. Adjustment for multiple comparisons: Bonferroni

**Fig 5:**
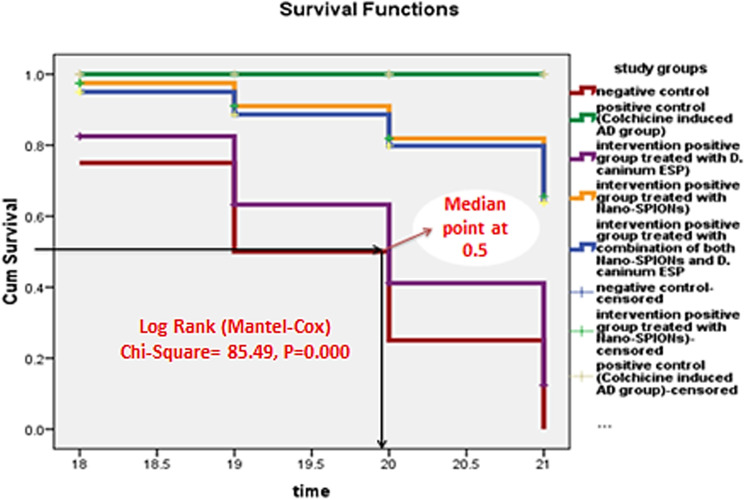
Displays the Log Rank (Mantel-Cox) Kaplan-Meier curve.

**Fig 5** displays the Log Rank (Mantel-Cox) Kaplan-Meier curve, which estimates the effects of intervention study groups based on the least amount of time compared to the negative control group and normal cognitive parameters after intervention by study drugs. The results show significant differences in time between groups (X2 = 85.49, P = 0.000) at df = 4 and 0.05 level of significance, with the least amount of time being nearly 20 days at the median point 0.5. Group III, treated with *D. caninum* ESP, has a lower area under the curve than the other intervention groups. Furthermore, the Kaplan-Meier curve indicated that Group III was close to the negative control group, so it was considered the fastest intervention drug compared to the other intervention drugs in the current study concerning cognitive parameters.

**Fig 6:**
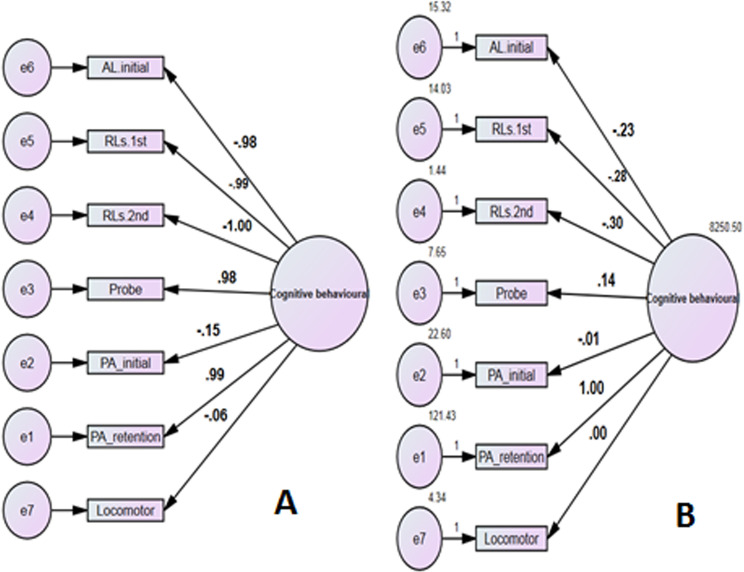
AMOS analysis to guess the relationship (standardized values) (A) and the regression (unstandardized values) (B) between the various physiological cognitive parameters.

In **Fig 6**, we can see how well the performance was judged by using AMOS analysis to guess the correlation r values (standardized values) (A) and the regression beta coefficient (B slope) (unstandardized values) between the various physiological cognitive parameters that were observed and the cognitive behaviors that were latent. These predictions show that the study found a strong positive correlation, r = 0.99, between effect step retention latency and cognitive behaviors. According to linear regression, an increase of one unit in effect step retention latency led to a corresponding increase in cognitive behaviors by one unit. This suggests that the effect of step retention latency is an effective factor in enhancing cognitive behaviors.

## Discussion

Bacteria use enzymatic reactions like oxidation, reduction, sorption, and chelation to produce nanoparticles as a defense against harmful heavy metals [45]. *Bacillus velezensis* SMR cells have been shown in this study that they can make two different types of SPIONs: Fe_3_O_4_ and Fe_2_O_3_. They do this by using the substrates FeCl_3_ and FeSO_4_․7H_2_O, respectively. Indeed, earlier investigations have reported the synthesis of magnetic nanoparticles using *Bacillus*. Rabani et al. [46] reported on the manufacture of iron oxide nanoparticles (IONPs) from *B. circulans* supernatant. The green-to-greenish-black coloration of the supernatant was due to surface plasmon resonance. Moreover, Fatemi et al. [45] reported that *B. cereus* strain HMH1 produced magnetic IONPs outside of cells. As a result, the O-H, Fe-O groups, (C-O for Fe_3_O_4_ and C = O for Fe_2_O_3_) on the surface of both types of SPIONs served as capping agents for the SPIONs we produced. Rabani et al. [46] conducted an EDX investigation in agreement with our findings, indicating that biomolecules stabilize the biosynthesized IONPs, primarily composed of Fe, O, and C (41.74%, 27.36%, and 11.39%, respectively). Our findings, which used VSM analysis to look into the magnetic properties of the SPIONs we made, clearly show that the materials’ magnetic properties change with size, which is in line with what Abdel Aziz et al. [47] said. In the 10–20 nm size range, SPIONs exhibit superparamagnetism, which has a linear relationship with size. The solitary magnetic domain of these nanoparticles acts as a “single super spin,” making them extremely magnetically responsive and quick to respond to magnetic fields. The XRD data obtained from our SPIONs are exactly the same as those reported by Singh et al. [48]. Their observations reveal an inverted spinel structure in magnetite, where Fe (III) ions distribute haphazardly in octahedral and tetrahedral sites, and Fe (II) ions in octahedral sites. It is also cubic crystalline, with reflection patterns of 220, 311, 400, 511, and 440.

Our most recent study showed that SPIONs might be responsible for the harmful effects on the tegument of cestodes after a 24-hour exposure. Cestodes may feed throughout the tegument, improving their integrity by expanding the absorption surface and reducing the effective time of these nanoparticles. Tegumental abnormalities include laceration, multiple white lesions, skin damage, sequential tucking and deformation of the epidermis, and the appearance of surface pores. Ruminants exposed to a methanolic preparation of *Balanites aegyptiaca* fruits showed tegument changes in samples of the trematode *Paramphistomum microbothrium*. These changes could result from increased efforts to spread and interchange the exterior tegumental membrane that the drug action has disrupted, as well as passive drug dispersion through the body wall [49,50]. Shalaby et al. [38] investigated the impact of fruits from the *Balanites aegyptiaca* plant on the tegument of adult *D. caninum* [38]. The results show that the *Balanites* extract affects adult worms in a cestocidal manner, mainly affecting the tegument. The principal modification of the extract’s effects, which depend on the length of exposure, is the deformation of the tegumental surface. Millán-Orozco et al. [51] conducted *in vitro* experiments to determine the effectiveness of Pyrantel-Oxantel in killing *Dipylidium caninum* tapeworms. The researchers cultivated adult tapeworms at varying concentrations in the medicine. The *D. caninum* cestodes showed a motility decline of 28% after one hour and 52% at the second, with a 100% mortality rate. Also, Pyrantel-Oxantel made the tegument thinner by 42.5%, which shows that the drug had a therapeutic effect on *D. caninum* by making the tegument thinner and increasing mortality. Justin et al. [52] stated that tissue damage from SPIONs significantly altered the external structure of *Eudrilus eugeniae*. We assume that the integration of SPIONs is the cause of the deteriorated circular muscles and the degradation of the superficial layer. Previous research has demonstrated that exposure to Fe_2_O_3_ inhibits earthworms’ growth and reproduction, while Fe_3_O_4_ nanoparticles cause their guts to disintegrate, fibrose, and erode.

The failure of disease-modifying therapies for AD is likely due to a variety of factors, such as mishandled drug dosages, improper target selection during treatment, delayed treatment initiation during AD development, and, most importantly, insufficient awareness of the disease’s intricate pathophysiology, which may require mixed therapies instead of monotherapy [53]. Fox and colleagues’ significant association between the prevalence of AD and hygiene [54] supported the hygiene argument. Therefore, we investigated the effects of colchicine on the *D. caninum* ESP and Nano-SPIONs in a rat model of AD. The results of passive avoidance and the modified water maze demonstrated enhanced memory consolidation in both treatment groups. The current data indicates that the Group V intervention positive group, which received treatment with Nano-SPIONs and *D. caninum* ESP, was the most successful intervention group. This was because its biochemical markers matched the negative control group.

In accordance with our discoveries, Sanati et al. [55] stated that low dosages of SPIONs reduced Aβ accumulation and improved cognitive function in the AD model. Conversely, large doses of SPIONs did not counteract the adverse effects of Aβ fibrillation on hippocampal protein expression and spatial memory. SPIONs had a significant impact on MDA levels in wild-type rats’ hippocampus. It was the main effect of SPIONs that was important for MDA levels in the hippocampus of rats that were given Aβ [55]. Hippocampal synaptic disruption is the underlying source of Aβ-induced cognition and information retention deficiency [56]. The applied magnetic field can transport SPIONs to the desired tissue and influence their behavior. There are two ways in which positively charged SPIONs impact the Aβ fibrillation process. By altering the concentration of monomeric proteins in solution, they impact the nucleation time. Moreover, SPIONs attach to proteins and modify their structure [28]. When we compared the negative control group to the treated group IV (Nano SPIONs), we found that the latter had the greatest statistically significant differences in terms of improving the cognitive parameters according to MWM, leading to a decrease in the spatial learning process. Additionally, these treated groups demonstrated a significant decrease in the first and second RLs, respectively, compared to the negative control group. Interestingly, compared to the negative control group, the treated groups with group V (the combination of SPIONs and *D. caninum* ESP) displayed the largest probes.

The study also found that the groups that were treated with Nano-SPIONs and their combination with *D. caninum* ESP had much longer step-through latency times before they stabilized, which is a good sign for memory consolidation. In line with what we found, Amanzadeh Jajin et al. [57] also found that quercetin-conjugated superparamagnetic iron oxide nanoparticles (QT-SPIONs) protect neurons from AD more effectively than free quercetin. When it came to learning and memory deficits, QT-SPIONs and the control group were almost identical. This group and the control group saw similar results. Consequently, the antioxidant activity of QT-SPION stopped the advancement of cognitive deficiency by preserving the equilibrium of antioxidant enzymes in the hippocampus tissues of AD model rats. In the MWM probe test, SPIONs had a significant effect on the average latency, distance traveled, and time spent in the desired quadrant during training days. The main effect of SPIONs was statistically significant for the average delay over different training days in MWM. On day 21 (P < 0.001), the MWM test showed that therapy with QT-SPIONs had to deal with a significant extension of escape latency caused by AlCl_3_. The AlCl_3_ C QT-SPION group also proved that the AlCl_3_ C QT groups and AlCl_3_ C SPIONs reduced escape latency and improved spatial memory [55]. Ebrahimpour et al. [58] and Amanzadeh et al. [59] also demonstrated the ability of QT-SPIONs to correct streptozotocin-induced memory and learning deficits using MWM and the shuttle box. Additionally, Sanita et al. [55] found that Bucla + SPIONs or low doses of SPIONs decreased spatial memory loss. Interestingly, many MWM training days demonstrated the positive effects of modest dosages of SPIONs. Depending on the specific chemical, protocol, and route of administration, the first day of training may influence the rats’ performance in the MWM task. Alterations in neurotransmitter discharge and action primarily cause this [60,61].

Iatrogenic helminth therapy has recently attracted interest, despite the fact that helminth infections undoubtedly harm the health of many affected vertebrate hosts [62,63]. Researchers have recognized the pharmacopoeic effects of helminths since the discovery of immunoregulatory proteins [64] and, more recently, metabolites [65,66]. This study is the first to link AD treatment to *D. caninum* severity and ESP efficacy. The ESP of *D. caninum* consists of a minimum of 12 small molecules (SM) that have well-documented bioactivities relevant to human health. Some important metabolic pathways that control inflammatory processes are docosahexaenoic acid, an unsaturated fatty acid that has been shown to reduce inflammation [67], acetate, a short-chain fatty acid that controls blood flow in the colon and ileal motility [68], and others. People are interested in treating inflammatory bowel diseases by getting the three main short-chain fatty acids (SCFAs) acetate, propionate, and butyrate to the gut. Because of this, one might think that getting infected with *D. caninum* could be a way to get these therapeutic SCFAs straight to the GI tract [69]. Moreover, the impact of inadequately documented cestode extracts on the immune system function of humans and rats in the context of autoimmune or allergic illnesses remains unclear. Research using cestodes, a type of helminth, has supported the idea that infection with these parasites can stop another disease from happening. However, problems still need to be solved regarding the removal of immunomodulatory molecules from the parasite, mixed pathogen infections, long-term effects, and possible side effects before helminths can be used as anti-inflammatory drugs for human diseases [70]. In order to decrease the amount of Aβ peptides produced by the brain, γ- and β-secretases have been manipulated as a management for Alzheimer’s [71]. Immunotherapy that targets Aβ has been widely used because it has been determined that Aβ collection in the brain is an early trigger for AD [72]. Long-term helminthic infection mimics other chronic disorders by down regulating the immune system, sparing the body from major harm [73]. During organ migration, helminths employ a variety of immunological regulatory mechanisms to ensure their survival and mitigate the potential harm from tissue damage to the host. Excreted proteins, extracellular vesicles, and metabolites are used to manipulate the immune defense of the infected host by influencing signaling mechanisms, hindering tissue repair, and stimulating or suppressing immune cells in order to modify the host’s environment in a way that is advantageous to them [74].

The current study found that rats treated with *D. caninum* ESP alone did not show the same progression of AD as rats treated with *D. caninum* ESP and Nano-SPIONs together. Drug-loaded Nano-SPIONs or Nano-SPIONs with enhanced drug delivery may account for this phenomenon. Stability, controlled drug release kinetics, high drug loading capacity, and drug encapsulation are among the characteristics of Nano-SPIONs. A series of ligands linked to the NPs’ surface can smoothly improve these properties [75,76]. Nano-particulate-based drug delivery techniques improve blood-brain barrier (BBB) transit. Their small size, altered surface, enhanced ability to dissolve, and capacity to interact with a range of biological processes set them apart. These intact NPs biodegrade and liberate the drug within the brain’s microenvironment [77].

The motoneuronal function of *D. caninum*, the immunomodulating molecule AChE, influences the production of cytokines and alters the response of macrophages. Helminths produce acetylcholine esterase (AChE), a factor that directly disrupts gene expression and intracellular signaling. Helminths generate AChE in various forms, demonstrating its diverse functions. AChE is required to preserve motility and manage the relationship between acetylcholine (ACh) and nicotinic acetylcholine receptors (nAChR). By acting as an ion channel and causing membrane depolarization in response to ion inflow in cells, Ach’s receptor regulates muscular contraction. Because AChE is essential to *D. caninum* motoneuronal activity, it limits this interaction and avoids overstimulation by cleaving ACh to choline and acetate [78]. In order to create IgE and IgG4 in humans or IgE and IgG1 in rats, the Th2 response subsequently stimulates immunoglobulin class switch recombination in B cells, principally through direct T-B cell contact and interleukin-4 (IL-4) receptor (IL-4R) (IL-4R/STAT6) signaling [78]. IgE antibodies have the ability to activate basophils involved in protective immunity after secondary helminth infection. Helminth infections also make the immune system weaker by increasing the number of Tregs (immunological regulatory monocytes) and the cytokines transforming growth factor beta (TGFβ) and interleukin 10 (IL-10) [79].

Despite the paucity of data in this area, existing knowledge lends credence to the hypothesis that studying helminth-rodent model systems can result in the progress of novel cures for inflammatory illnesses [70]. Wu et al.‘s research [80] suggests that ESPs derived from larval *Echinococcus granulosus* play a unique role in counteracting the cognitive decline resulting from obesity. They accomplish this by strengthening the gut-brain axis, which in turn stimulates astrocytes and microglia while reducing neuroinflammation. Whether ESPs can directly cross the blood-brain barrier and reduce neuroinflammation in the hippocampus and prefrontal cortex is currently uncertain. These results, which show down regulated pro-inflammatory cytokine expression and decreased macrophage activation and proliferation in the colon of rats fed a high-fat diet, align with previous studies that demonstrated the down regulation of immunological effects mediated by extracellular soluble polymer (ESP) [81–82]. Finally, the current study must overcome several obstacles and limitations. First, the study will take longer than 21 days to finish because it is an experimental longitudinal study. Using the Log Rank (Mantel-Cox) Kaplan-Meier curve for accurate results was especially important for cognitive function. However, this was not allowed in animal houses where rats used in experiments were given standard medical care. The second problem was that the cognitive function analysis was done using an estimated seven parameters, making it exceedingly difficult to predict the normal cognitive function of the rat when the Alzheimer’s therapy medications, such as *D. caninum*-ESP or Nano-SPIONs, were administered.

## Conclusion

The current results concluded a link between reductions in biochemical biomarkers and Alzheimer’s pathology. In particular, the combination therapy was better at lowering levels of Aβ1–42, NF-kβ, and MDA while raising levels of SOD compared to the negative control group. Additionally, the use of Nano-SPIONs significantly improves cognitive functions compared to other intervention drugs. Lastly, the group treated with *D. caninum* ESP showed a faster recovery from Alzheimer’s disease, as evidenced by the lowest Kaplan-Meier survival curve at the median point.

## Supporting information

S3 Fig Size distribution data and curves of Fe_3_O_4_ and Fe_2_O_3_. *Bacillus velezensis* SMR cells (A) produced a mixture of SPIONs (Fe_3_O_4_ and Fe_2_O_3_) as black powders (B). The size distribution curve for Fe_3_O_4_ and Fe_2_O_3_ showed that they were mostly 5 and 6 nm in size (C and D), respectively.
(ZIP)

S5 Fig Log Rank (Mantel-Cox) Kaplan-Meier curve. Displays the Log Rank (Mantel-Cox) Kaplan-Meier curve.
(XLSX)

S6 Fig AMOS analysis. AMOS analysis to guess the relationship (standardized values) (A) and the regression (unstandardized values) (B) between the various physiological cognitive parameters.
(XLSX)

S1 Table Characterization. The biosynthesis and characterization of superparamagnetic iron oxide nanoparticles (SPIONs).
(ZIP)

S2 Table Discrimination analysis. Demonstrates the discrimination analysis of biochemical parameters in different brain tissues.
(PDF)

S3 Table MANOVA test. Shows the biochemical parameters changes according to the intervention study groups.
(XLSX)

S4 Table Multiple comparisons Bonferroni. Displays the pairwise comparisons of biochemical parameters among different study groups compared with the negative control group (reference group).
(XLSX)

S5 Table MANOVA test. Shows the cognitive parameters of MWM changes according to the intervention study groups.
(XLSX)

S6 Table Multiple comparisons Bonferroni. Presents the pairwise comparisons of cognitive parameters of MWM among different study groups compared with the negative control group (reference group).
(XLSX)

S7 Table MANOVA test. Shows the cognitive parameters of passive avoidance (PA) changes according to intervention study groups.
(XLSX)

S8 Table Multiple comparisons Bonferroni. Displays the pairwise comparisons of cognitive parameters of passive avoidance (PA) among different study groups compared with the negative control group (reference group).
(XLSX)
