# Supplementary material for: An experimental study of the dual modulation of the colchicine-induced rat model of Alzheimer’s disease by superparamagnetic iron oxide nanoparticles (SPIONs) and the soluble product of Dipylidium caninum adult worm
Source: PLoS One. 2025 Jun 3;20(6):e0324191. doi: 10.1371/journal.pone.0324191 (PMC12132939; doi:10.1371/journal.pone.0324191)
Supplement: S1 Table — The biosynthesis and characterization of superparamagnetic iron oxide nanoparticles (SPIONs). (ZIP) [file pone.0324191.s004.zip › S1 Table/S1 Table EDX (Fe3O4).pdf]

Electron Microscope Unit
Faculty Of Science
Alexandria University

Sem_SED_006

Signal SED
Landing Voltage 20.0 kV
WD 10.0 mm
Magnification x500
Vacuum Mode HighVacuum

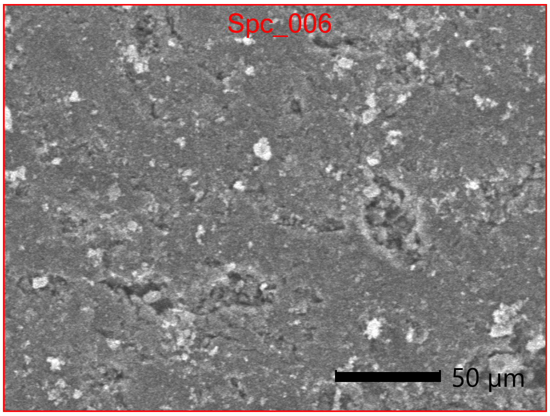

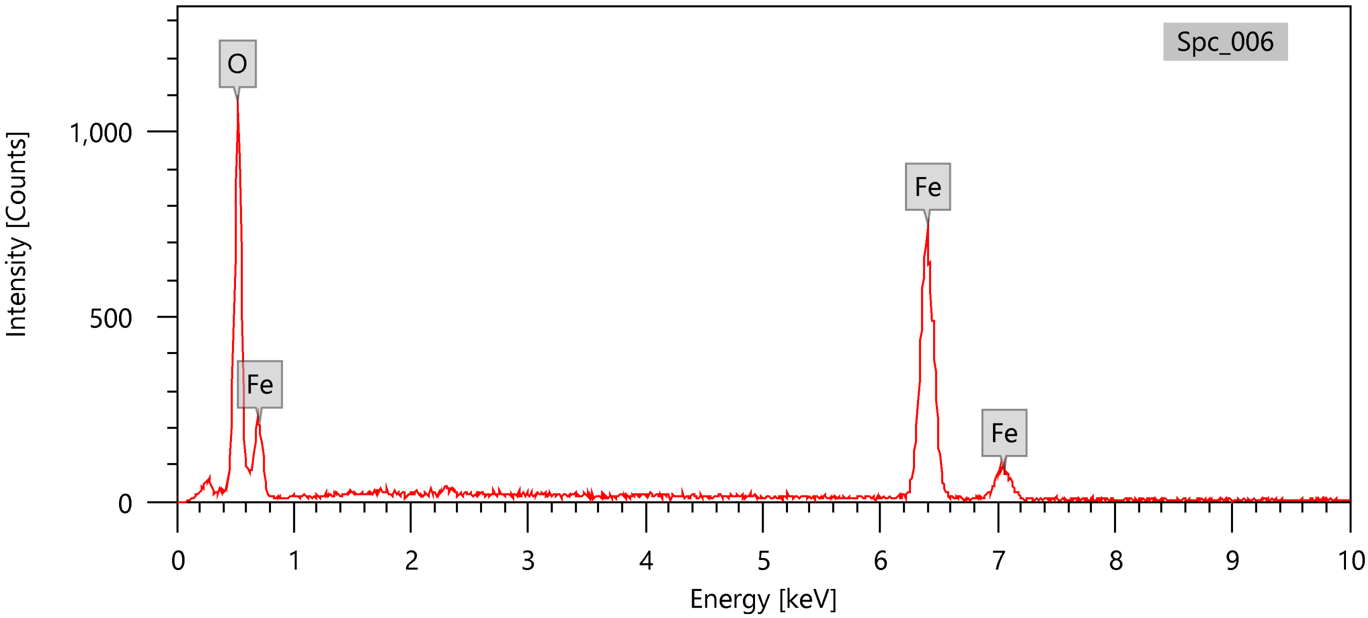

Items	Value
measurement conditions	
Acceleration voltage	20.00 kV
Probe current	-
Magnification	x 500
Process time	T2
Measurement detector	First
Live time	30.00 seconds
Real time	30.08 seconds
Dead time	0.00 %
Count rate	1164.00 CPS

Display name	Standard data	Quantification method	Result Type
Spc_006	Standardless	ZAF	Metal

Element	Line	Mass%	Atom%
O	K	34.07±0.40	64.34±0.76
Fe	K	65.93±0.86	35.66±0.46
Total		100.00	100.00
Spc_006			Fitting ratio 0.0397
